# “Do they REALLY trust us”?: Lessons from a volunteer research registry

**DOI:** 10.1017/cts.2024.584

**Published:** 2024-11-11

**Authors:** Sylk Sotto-Santiago, Sarah Wiehe, Brenda Hudson, James Slaven, Conor Vinaixa, Rebecca Bruns, Gina Claxton, Lynsey Delp, Dustin Lynch, Sharon Moe

**Affiliations:** 1 Department of Medicine, Indiana University School of Medicine, Indianapolis, IN, USA; 2 Indiana Clinical and Translational Sciences Institute, Indianapolis, IN, USA; 3 Department of Pediatrics, Indiana University School of Medicine, Indianapolis, IN, USA; 4 Department of Biostatistics and Health Data Science, Indiana University School of Medicine, Indianapolis, IN, USA; 5 Medical Education, Indiana University School of Medicine, Indianapolis, IN, USA

**Keywords:** Trust, trustworthiness, registry, diversity, clinical trials

## Abstract

**Background:**

All IN for Health is a well-established community-academic partnership dedicated to helping improve the lives of Indiana residents by increasing health research literacy and promoting health resources, as well as opportunities to participate in research. It is sponsored by the Indiana Clinical and Translational Science Institute (I-CTSI). The study’s purpose was to measure trust in biomedical research and healthcare organizations among research volunteers.

**Methods:**

The Relationship of Trust and Research Engagement (RTRE) survey was developed utilizing 3 validated scales. The RTRE consisted of 36 items in a 5-point Likert scale with three open-text questions. We conducted 3 focus groups with a total of 24 individuals ahead of the survey’s launch. Recruitment was done through the All IN for Health newsletter. The survey was administered in the summer of 2022.

**Results:**

Six hundred and sixty-three individuals participated in the survey. Forty-one percent agreed that doctors do medical research for selfish reasons. Moreover, 50% disagree that patients get the same medical treatment regardless of race/ethnicity. Sixty-seven percent think it is safe to participate in medical research, yet 79% had never been asked to participate. Ten percent believe that researchers select minorities for their most dangerous studies and expose minoritized groups to diseases.

**Conclusion:**

The utilization of tools to measure trust will facilitate participant recruitment and will assist institutions and investigators alike in accountability. It is imperative, we work toward understanding our communities’ trust in medical research, assessing our own trustworthiness, and critically reflect on the authenticity of our efforts.

## Introduction

Trust is essential to community engagement and biomedical research. Building and maintaining mutually respectful relationships is at the very core of this work. Wilkins et al. defined trust as a multidimensional construct that many people find difficult to define. In general, trust refers to a firm belief in the reliability, truth, and ability or strength of someone or something. (p. S6) [1]. Trust is also considered as a variable concept affected by the individuals in a particular partnership. Individuals are unique in their willingness to trust, often influenced by their lived experiences [2]. Indeed, during discussions about trust, we have asked individuals how they would define it. Often, it is a very personal and subjective answer, yet most seem to agree that several values and concepts when placed together define trust, such as honesty, respect, reliability, safety, and protection, among others [3–5]. One definition of trust speaks to allowing oneself to become vulnerable to potential exploitation by a person. Trust involves the risk that people we trust will not pull through for us [6]. In the context of participants and researchers, it means that researchers have designed and conducted the research with good intentions and will toward them; trust that researchers champion their health above everything else. This also highlights trust as a relational matter. Scoping reviews have also shown trust as a relational concept, involving a dyadic relationship where trust is being given by a trustor and received by a trustee [2]. Voluntariness is also described as being a characteristic of trust in relationships as it cannot be demanded or given freely [6].

Trust in biomedical research can also be multifaceted. Several factors influence an individual’s level of trust in research including culture and beliefs, educational attainment, personal and family diagnoses, experience navigating healthcare systems, and what they know about their cultural identity and communities’ experiences with research. Scholars and practitioners in community engagement have provided frameworks and resources emphasizing the importance of building trust. For example, Dave et al. proposed a five-cluster solution consisting of: authentic, effective, and transparent communication; mutually respectful and reciprocal relationships; sustainability; committed partnerships; and credibility and methodology to anticipate and resolve problems [7]. At the very foundation, practitioner scholars reemphasize the importance of effective communication throughout so that communities understand the process, goals, and intended outcomes, along with key information to make an informed decision [8].

Despite this emphasis and shared strategies, we are delving deeper into trust among patients, research volunteers, and community members who are involved in research [1]. Because trust takes time, it also remains elusive even after decades of community engagement work, and we do not have much explanation other than one of its many challenges is its complexity and a clear record of historical trauma in relation to research atrocities [1,7].

Trustworthiness has also been hard to define, but in our interactions with the public, trustworthiness has been linked to similar values, such as honesty and reliability, with the critical aspect of following through in our commitments [3–5]. Trustworthiness is often referred to the propensity to fulfill others’ expectations regarding a particular action.

Scholars have also found that guilt-proneness is a better predictor of trustworthiness [9]. Based on this relationship between guilt and trustworthiness, researchers should try their best to avoid wrongdoings and admit their guilt and complicity in our current healthcare system; owning the past research atrocities could play a factor in becoming trustworthy. As associated concepts, we further discuss trust and trustworthiness as central to our conceptual framework.

### Trust in science

The public engagement and belief in science has eroded to new levels in a post-pandemic era. Americans’ trust in science and medicine is now below pre-pandemic levels. According to the Pew Research Center, in 2021, only 29% of US adults say they have a great deal of confidence in medical scientists to act in the best interests of the public, down from 40% who said this in November 2020. Similarly, the percentage with a great deal of confidence in scientists to act in the public’s best interests is down by 10 percentage points, to 29% [10]. Moreover, across racial and ethnic groups, confidence in medical scientists declined among white and Black adults over the past year, and it is more pronounced among White adults. Generally, confidence in scientists tends to track closely with confidence in medical scientists [11].

Why is this? Medicine and science face a credibility crisis that threatens its ability to protect people’s health. The public has been overwhelmed by too much information, growing polarization, disinformation campaigns by domestic and foreign entities, a media environment that rewards outrage, and the increasingly public nature of scientific research [11].

In community-academic research partnerships, a diverse group of stakeholders collaborate for the purpose of sharing mutually beneficial research objectives. Trust is considered a must in community-academic research partnerships yet, there are limited measures of the trust within the context of such partnerships. At the time of this study, a literature review yielded very limited number of scales. However, three scales appear highly relevant. Hall et al. developed the trust in medical researchers scale. Survey items were developed based on a conceptual model of the primary domains of researcher trust: safety, fidelity, honesty, and global trust. The authors concluded that trust in medical researchers can be a measurable single-factor construct including trust in safety, researcher fidelity, and honesty [12]. Mainous et al. offered a tool for identifying individuals or groups of individuals who are unlikely to participate in medical research with their patient trust in medical researchers [13]. Shea et al. developed the distrust in healthcare organizations scale to assess two primary domains of distrust: values and competence. They posit that the scale provides an understanding of what types of interactions are most potent for changing reported levels of distrust [14]. All of these scales focus on trust and contribute toward identifying community-specific concerns about researchers and recruitment efforts even among historically minoritized populations. Promising scales are being developed, and Stallings et al. offer a greater understanding of trust in research among marginalized populations, incorporating trust dimensions of secrecy, fairness, community benefit, and privacy [15]. Trust measures are critical in understanding and facilitating strategies to amplify trust and trustworthiness of research.

Another important observation is the focus on trust, mistrust, and distrust among these measures and throughout the literature. For our study, we define mistrust as a cautious attitude toward others with a careful and questioning mindset about the trustworthiness of the other and information. We define distrust as a belief that an individual is not trustworthy or that the source of information is intentionally misconstrued [16,17]. In the context of our study mistrust and distrust can be influenced by historical and contemporary factors that vary by individual and their identity [17].

Our study’s purpose was to measure trust in biomedical research and healthcare organizations among individuals in our research registry and evaluate how we may improve recruitment as an organization. In this study, we introduce All IN for Health, an initiative of the Indiana Clinical and Translational Science Institute (I-CTSI). All IN for Health is a well-established community-academic partnership dedicated to helping improve the lives of Indiana residents by increasing health research literacy and promoting health resources, as well as opportunities to participate in health research and clinical studies.

All IN for Health maintains a research volunteer registry that is available to all Indiana Clinical and Translational Sciences Institute (CTSI) investigators. This registry was developed in 2011 under the name of INresearch.org and changed to All IN for Health in 2017. The goal of the volunteer registry was to reach a large number of Indiana residents interested in participating in research at various institutions affiliated with the Indiana CTSI: Indiana University, Indiana University School of Medicine, Purdue University, University of Notre Dame, and the Regenstrief Institute. The registry is one of the free resources provided by the Indiana CTSI. Currently, there are 14,463 volunteers registered. These volunteers have been recruited via the All IN for Health digital presence, as well as from health care clinics, health fair events, health provider websites, and other media sources. Registrants complete a consent, create an individual profile, and provide health information including demographics, health conditions, and medication usage. This information is used to link the registrants with investigators and their studies and develop a recruitment cohort of participants. We have also been able to link 76% of the volunteers to an electronic health record.

### Conceptual framework

Trust is the central conceptual framework of this study. In the context of All IN for Health as an institutional initiative, we define trust as the belief that an institutional entity will act in a communities’ best interest; the belief by research volunteers that their well-being is of utmost importance and considered before the interests of the study or the researcher. We also define trustworthiness in the context of this community-academic partnership as a trustworthy partnership where the community bestows trust. This definition is informed by two constructs, the Association for American Medical Colleges (AAMC) principles of trustworthiness and the concept of deserved trust [18]. AAMC principles of trustworthiness features ten principles inspired by community members’ insights into how academic medical centers can demonstrate they are worthy of their community’s trust with trustworthiness considered as a quality an individual demonstrates making them worthy of confidence, making them responsible and safe [18,19]. Deserved trust considers what it is that the biomedical research community should be trusted to do and ensuring this trust is deserved rather than expected or misplaced [20]. Hence, both constructs highlight the importance of trustworthiness as an honor conferred.

Additionally, we include a critical lens as a conceptual consideration. One that questions how problems are defined and acknowledges that assumptions exist in the context of social systems. When we use critical theories to examine current issues in health and biomedical research, we acknowledge external influences and become able to critique and examine situational and historical forces causing health disparities and inequities. One of the most visible successes, and not seen as part of a critical theory movement, is the appreciation and emphasis of structural and social determinants or drivers of health, a perspective that was once underdiscussed and is now dominating medicine and public health.

## Methods

### Instrument design

The Relationship of Trust and Research Engagement (RTRE) survey, as it was titled, included three validated surveys: Healthcare System Distrust, Trust in Medical Researchers scale, and Patient Trust in Medical Researchers [12–14]. The Healthcare System Distrust scale was designed to consider multiple issues related to HCO distrust and what types of interactions with the health care system are most relevant for changing levels of distrust [12]. The Trust in Medical Researchers’ scale is a measurable single-factor construct including trust in safety, researcher fidelity, and honesty [13]. Lastly, the Patient Trust in Medical Researchers was developed to assess trust in medical researchers acknowledging that mistrust is a barrier to research participation [14].

The research team and individuals consulted for this study include qualitative, quantitative, and mixed methods researchers, survey design experts, community-engaged scholars, a biostatistician, and medical students. As a result, the scales were unified in one survey. The RTRE included 9 items from the Healthcare System Distrust scale, 12 items from the Patient Trust in Medical Research, and 15 items from the Trust in Medical Research Scale. RTRE was finalized as a 36-item survey, 5 point Likert scale that also included open-text questions: “Are there any previous experiences that made you distrust medical research?,” “What would make you more comfortable to participate in research?,” and “How do you feel Indiana University and School of Medicine, Purdue University, and University of Notre Dame should connect with the public when it comes to medical research?” Along with the Regenstrief Institute, these 3 academic institutions are partners in the I-CTSI.

As the RTRE survey is a combination of validated scales, we conducted 3 focus groups for cognitive interviews with a total of 24 individuals to confirm the final survey fulfilled its intended purpose. Participants were recruited via email invitations sent to medical students, student interest groups, and word of mouth with All IN for Health advisory board members. Participants represented similar educational background, gender, race, and ethnicity as All IN for Health’s registry. As a method, cognitive interviewing is an evidence-based, qualitative method designed to investigate how individuals process and respond to survey questions. Group cognitive interviews offered meaning and thought processes by respondents which involved comprehension, retrieval of information, judgment, and response selection. In addition, they have been reconceptualized as the background social context that may influence how well questions meaningfully capture the life of the respondent [14]. The interviews prompted updating and clarifying of some terms used in survey questions. These changes did not impact the validity of the scales or responses and were simply to improve the clarity of the scales. The language was minimally changed in the RTRE compared to the original scale item. Table 1 summarizes these changes.

**Table 1.** Language changes between scale and survey

Item	Modification
**Hall et al. (2006) Trust in Medical Researchers**	
*A* doctor would never ask me to be in a medical research study if the doctor thought there was any chance it might harm me	*My* doctor would never ask me to be in a medical research study if the doctor thought there was any chance it might harm me
**Mainous et al. (2006) Patient Trust in Medical Researchers**	
Medical researchers act differently toward minority subjects than toward white *subjects*	Medical researchers act differently toward minority participants than toward white *participants.*
Some medical research projects are secretly designed to expose minority groups to diseases such as AIDS.	Some medical research projects are secretly designed to expose minority groups to diseases such as AIDS
Usually, medical researchers tell participants everything about possible dangers. All in all, medical researchers would not conduct experiments on people without their knowledge	This item was modified as two items: Usually, medical researchers tell participants everything about possible dangers. Medical researchers would not conduct experiments on people without their knowledge.
Researchers are more interested in helping their careers than in learning about health and disease.*	*Medical* researchers are more interested in helping their careers than in learning about health and disease.
**Shea et al. (2008). Health Care System Distrust Scale**	
Health Care System	Focus group participants encouraged the use of *health care organization* instead of health care system.
Patients get the same medical treatment from the Health Care System, no matter what the patient’s race	Patients get the same medical treatment from healthcare organizations, no matter what the patient’s race *or ethnicity.*

To note, mistreatment and provider bias experiences were shared by interviewees more specifically during the question: “Medical researchers act differently toward minority participants than toward white participants.” Participants spoke about a perceived “lack of interest in truly recruiting minorities into studies,” “they [medical researchers] don’t meet us where we are,” “if I am discriminated during an appointment, why would I think medical research would be different”? And “all of us know what happened during COVID-19 and to the Black doctor.” Interviewees also responded positive when asked about their opinions about local institutions of higher education and the academic health center with respect to types of research, reputation, and credibility.

### Data collection

The study was advertised in the All IN for Health newsletter which has over 30,000 subscribers, and an invitation was sent to 13,800 volunteers in the registry at the time of survey administration. The invitation provided a description of the study with a direct link to the electronic survey (REDCap). The study took place during the summer of 2022. The Indiana University IRB approved this research study.

### Data analysis

Chi-Square tests were performed to determine if there was significant heterogeneity between historically marginalized racial group participants and white participants for each of the RTRE survey items (JS). Historically marginalized groups were defined in this study as those who have been historical marginalized and minoritized by society regarding health care access and health outcomes. For this data set, this term included Black/African American, Hispanic/Latine, and Asian/Asian American/Pacific Islander. Due to small cell sizes for some of the responses, it was decided to dichotomize each question into agree vs. disagree/neither. Analyses were performed using SAS v9.4 (SAS Institute, Cary, NC). For the qualitative analysis, we used an inductive approach to generating codes and themes and analyzed data using the constant comparative method, in which essential concepts were coded and compared over time to extract recurrent themes. Three authors independently read responses to generate codes, also creating thematic categories. We met to discuss data interpretation as a group (SSS, CV, RB).

### Research team positionality

Collectively, we are community-engaged and equity-minded scholars. Our knowledge of our fields of study and practice informs how we understand this data and relate to these results. We believe the study of trust in biomedical research can also generate honest conversations about health equity and inclusive research in addition to creating better practices for advancing health equity through intentional and deliberate efforts in equitable recruitment. It is from this lens that we offer a critical lens for conceptual consideration along with critical reflexivity of these findings. It is from this perspective that we offer a critical lens for conceptual consideration along with critical reflexivity of these findings. Moreover, as a majority female and White research team, we practice cultural humility in seeking understanding about health inequities and systems of oppression. We invite the readers and communities to join in conversation as a critical aspect of trust.

## Results

**Figure 1.**
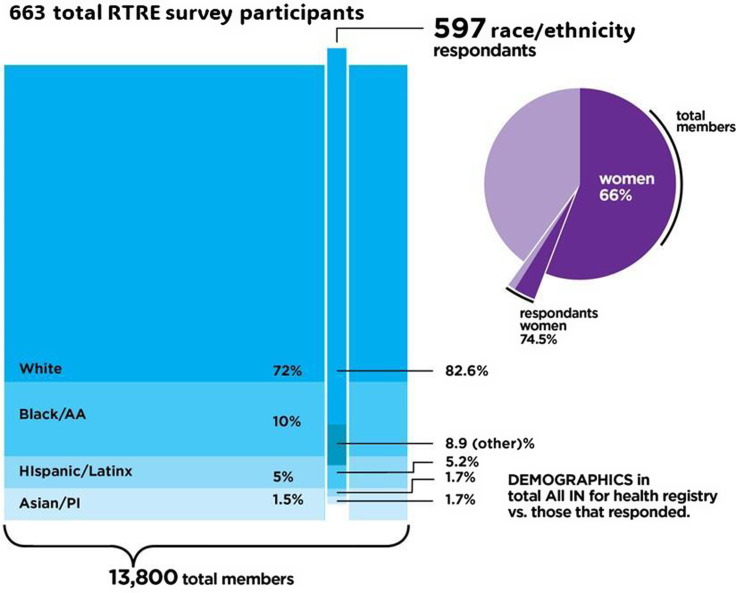
Participant demographics. AA = Black/African American; PI = Asian American/Pacific Islander; RTRE = Relationship of Trust and Research Engagement.

The RTRE was distributed to 13,800 volunteers in the All IN for Health registry, of which 663 answered the call to participate in the survey (Figure 1). However, 597 individuals answered race/ethnicity demographic questions. The demographic sample includes: White, which constitutes 72% of the registry vs. 82.6% of respondents; Asian/Asian American/Pacific Islander identified groups, 1.5% in the registry, and 1.7% of respondents. Black/African American and Hispanic/Latine were not as representative of the registry at low rates of participation (5.2% and 1.7%, respectively). Individuals selecting “other” as a category represented 8.9% of participants.

Results indicate that 48.2% of participants were over the age of 60 and 74.5% respondents were women. An estimated 32.6% had completed a bachelor’s degree, and 43.0% made a salary of less than $75,000 per year.

Forty-nine percent believed that healthcare organizations (HCOs) covered up their mistakes. Fifty-nine percent believed that HCOs put money above patients’ needs and that 41% of doctors do medical research for selfish reasons. Moreover, 50% disagree that patients get the same medical treatment regardless of race and/or ethnicity. Sixty-seven percent think it is safe to participate in medical research, yet 79% had never been asked to participate in medical research by their doctor. Concerning race and ethnicity, close to 30% think that medical researchers act differently toward minority participants. Ten percent believe that medical researchers select minorities for their most dangerous studies and some medical research projects are secretly designed to expose minoritized groups to diseases.

In general, participants suggested what would make them more comfortable in participating in medical research: if they had a conversation and were asked by their doctor, and if the institutions involved had better education programs and public messaging about the importance of medical research and participation. Regarding how institutions should connect with the public when it comes to medical research, responses suggest better coordination between these institutions, education about the importance of medical research, and the responsibilities of institutions to advocate for healthcare access. Lastly, regarding previous experiences that have contributed to their potential distrust in medical research, responses point out to historical atrocities in research ethics, such as Tuskegee. In addition, they pointed to the COVID-19 pandemic’s media coverage, racism in the healthcare system, and misinformation widely available. Table 2 summarizes a sample of statements.

**Table 2.** Qualitative analysis sample statements

Question	Open-text responses
Are there previous experiences that made you distrust medical research?	*-Hearing about historical abuses of minorities in medical research.* *-Tuskegee* *- Tuskegee- not personal experience, obviously, but extremely troubling* *-Based on the history of African Americans, distrust is inherent.* *-Not personally, but how about Tuskegee, Nazis, etc.* *-theories of COVID-19 contraindicating each other when it first came out* *-Everything about COVID-19* *-Personal experience* *-Seeing family members having surgery/tests/treatments that they do not need.* *-My mother’s medical information was shared for research purposes without her knowledge or consent* *-Only dishonest research(ers) that I have read about* *-not personal experience, I have participated in many research studies over the course of the past 5 years. However, the global pandemic and the behavior of Anthony Fauci and WHO have made me distrust much of what we were told about vaccines and the harm caused by the virus.*
What would make you more comfortable to participate in research?	*-Having a conversation with my doctor prior to participating.* *- If it was recommended to me by someone I personally know and trust, like my doctor.* *-to have my provider know about current studies that I could participate in* *-As much information as possible before starting the study and in-person conversations with the doctors conducting the research studies.* *-Going over all the information with the doctor, hearing about other peoples’ experiences in the study.* *-Additional educational resources about the topic of research and ways my participation may help people in the future* *-Health-literacy literature on topic provided to participants and opportunities to ask questions* *--Education. Stories, interviews, etc. on physician office TV’s. I’ve seen postings in cancer clinic areas for those patients but not in general waiting areas for the public.* *-Knowing who is funding the research and the degree to which my participation directly benefits the researcher.* *-Female researchers*
How do you feel Indiana University/School of Medicine, Purdue University., and University of Notre Dame should connect with the public when it comes to medical research?	*- Coordinate and share information and results* *-Tell the TRUTH* *-They should be very transparent about how research is funded and how that affects the researchers’ behavior toward patients. They can also emphasize how participants in research have helped tangibly improve medical care.* *-I think that more focus on HOLISTIC health would be massively beneficial to people. Many health problems cannot be solved by medicine alone, we need safe streets where kids and adults can exercise, healthy food programs, mental health services. This would all make people better all around, and then if they need medicine it should be AFFORDABLE - meaning that the medical communities at these schools should be lobbying for UNIVERSAL healthcare and for Big Pharma to stop price gouging the citizens of the US.* *-let people know when their contributions have contributed to research* *-Provide general public education on different types of studies, what those types of research studies entail, who is involved (various staff and their job descriptions), how privacy and safety are maintained, etc. This could be done as a public service style format, or a series of interviews (staff and human interest stories) or other formats as suggested by media professionals.* *- increased social media exposure would be beneficial in attracting younger populations and a more obvious presence within doctors’ offices/health clinics could attract older adults and/or more specific patient populations* *-advertise more, share stories of medical research and how it led to treatment or diagnosis for more people, sharing good outcomes and highlights* *advertise new, needing people for research events. --come to the masses through local news request.* *-More publicity on ongoing research.*

Data analysis was also performed to examine differences in survey responses by the race/ethnicity of participants (Table 3). Several statements reached statistical significance in the difference between white and historically marginalized groups. For the following statements, the marginalized groups reported less trust in medical research: it’s safe to be in a medical research study; most medical researchers would not lie to people to try to convince them to participant in a research study (*p* = .0009); some medical research projects are secretly designed to expose minority groups to diseases (*p* < .0001); to get people to take part in a study, medical researchers usually do not explain all of the dangers about participation (*p* = .0275); medical researchers act differently toward minority participants than toward white participants (*p* = .0005); and patients get the same medical treatment from healthcare organizations, no matter what the patient’s race or ethnicity (*p* = .0224).

**Table 3.** Responses by race/ethnicity

Item	Historically marginalized groups; *n* = 70 (12.5%)	White *n* = 492 (87.5%)	*p*-value
My doctor would not ask me to be in a medical research study if they thought there was any chance it might harm me Strongly Agree/Agree Disagree/Strongly Disagree/Neither	48 (68.6) 22 (31.4)	368 (75.3) 121 (24.7)	0.2306
Medical researchers have no selfish reasons for doing research studies Strongly Agree/Agree Disagree/Strongly Disagree/Neither	16 (22.9) 54 (77.1)	138 (28.1) 353 (71.9)	0.3573
My doctor has never asked me to be in a medical research study Strongly Agree/Agree Disagree/Strongly Disagree/Neither	53 (75.7) 17 (24.3)	392 (79.8) 99 (20.2)	0.4256
It’s safe to be in a medical research study Strongly Agree/Agree Disagree/Strongly Disagree/Neither	42 (60.0) 28 (40.0)	377 (77.1) 112 (22.9)	**0.** **0020**
Most medical researchers would not lie to people to try to convince them to participant in a research study Strongly Agree/Agree Disagree/Strongly Disagree/Neither	46 (65.7) 24 (34.3)	406 (82.5) 86 (17.5)	**0.** **0009**
Some medical research projects are secretly designed to expose minority groups to diseases Strongly Agree/Agree Disagree/Strongly Disagree/Neither	18 (26.1) 51 (73.9)	29 (5.9) 461 (94.1)	<**0.** **0001**
To get people to take part in a study, medical researchers usually do not explain all of the dangers about participation Strongly Agree/Agree Disagree/Strongly Disagree/Neither	13 (18.6) 57 (81.4)	48 (9.8) 442 (90.2)	**0.** **0275**
Health care organizations make too many mistakes Strongly Agree/Agree Disagree/Strongly Disagree/Neither	18 (25.7) 52 (74.3)	109 (22.3) 380 (77.7)	0.5225
Medical researchers act differently toward minority participants than toward white participants Strongly Agree/Agree Disagree/Strongly Disagree/Neither	32 (45.7) 38 (54.3)	125 (25.6) 364 (74.4)	**0.** **0005**
Patients get the same medical treatment from healthcare organizations, no matter what the patient’s race or ethnicity Strongly Agree/Agree Disagree/Strongly Disagree/Neither	13 (18.6) 57 (81.4)	157 (32.0) 334 (68.0)	**0.** **0224**

## Discussion

To our knowledge, this is the first study combining Healthcare System Distrust, the Trust in Medical Researchers scale, and Patient Trust in Medical Researchers [12–14]. Per the goals of this study, the trust scales offered an effective way to measure trust in biomedical research and HCOs. In addition, the resulting survey, the Relationship of Trust and Research Engagement Survey provided a unique perspective of individuals whose trust in research was enough to willingly join such registry. Based on the extant literature on community engagement and emerging literature on trust, we concur that this type of survey and validated scales can help identify strategies to improve recruitment within the communities we serve. Moreover, the results show remarkable differences and perspectives between historically marginalized groups and white counterparts as it pertains to trust in research. In what follows, we discuss these results in the context of its major findings: participation, education, and trustworthiness.

### Ask them to participate!

When the data are disaggregated, they demonstrate that 60% of participants from historically marginalized groups and 77% of white participants think it is safe to participate in research, yet a total of 79% had never been able asked to participate. Furthermore, 43% believe that their doctor/provider would not ask them to participate in a study if they thought there was any chance it may harm them.

Hence, what are the barriers for providers, especially in primary care, to encourage participation? This is an important concern because scholars have shown five crucial factors in clinical trial recruitment by providers: representing the importance of clinical trial recruitment in a providers’ professional identity, clinic-level interventions to facilitate referral, patient-related barriers, concerns about patient health management, and general knowledge gaps [21]. These results contribute to dismantling these barriers with encouraging data from the public and those willing to participate in research. In addition, the research infrastructure has long critiqued the barriers to research referrals and these data reinforce the importance of strong referral and provider education networks in the recruitment of research participants.

In the USA, those that often face the greatest health challenges are not adequately represented in health and clinical research studies. There has been limited progress in the last 30 years to increase participation of racial and ethnic minoritized populations, although the imperative is greatly known. This underrepresentation may lead to lack of access to effective medical interventions, compounds health disparities, and costs hundreds of billions of dollars according to the National Academies of Sciences, Engineering, and Medicine [22]. Their report affirms that health equity requires far more than equitable representation in biomedical research and risks not addressing health disparities and reinforcing more health inequities. Furthermore, our study, as does the National Academies, affirm that historically marginalized populations are willing to participate if asked [23]. Distrust and mistrust are not necessarily associated with a lack of willingness to participate, but other factors appear to be more of decisive factors as presented here.

### Importance of education

Findings highlight three important aspects. First, the perception of limited involvement by healthcare providers. The importance of provider education as it pertains to research opportunities, participation, and engagement of patients is essential. Overcoming this barrier is the first step toward patient accessibility to clinical trials and other forms of research.

Second, we need to examine opportunities for education from institutions and institutional accountability. What are institutions and partnerships doing to better educate the public about research? Most participants trusted the academic institutions (Indiana University, Purdue University, and Notre Dame) as institutions of high credibility in the state. However, it does not appear that institutional involvement nor investigators strongly highlighted their relationship to institutions and/or community partnerships.

Lastly, the COVID-19 pandemic left a huge gap in public trust due to misinformation and disinformation. What are we doing now to ensure this does not repeat itself? This aspect tackles the responsibility of institutions such as academic health centers to be part of facts and truth-campaigns. Participants referred to institutional accountability in ensuring that the institution’s voice is heard amid disinformation campaigns as institutions they trust. Trust in science needs to be addressed now before the next wave of attacks and discredit. Academic centers and health care systems can be part of this process based on their credibility within the communities they are meant to serve. These actions make us trustworthy. The public should continue to support research if institutions create opportunities to promote the public’s informed trust [24].

### What makes us trustworthy?

Although the study centered on trust, trustworthiness is its undercurrent. Earlier, we defined trust as the belief that an institutional entity, such as ours, will act in the community’s best interest and defined trustworthiness as the act when participants bestow trust on us. As researchers and supporters of research advancing health equity and well-being of communities, part of our call is to make ourselves trustworthy. How do we achieve that? The qualities associated with trust, such as honesty, respect, offering protection, and safety described earlier on are not simply innate, there is an opportunity to develop individuals, such as researchers, and learn from what the communities value. Professional development programs sponsored by institutions and communities can build skills, but also enhance opportunities to share kindness, empathy, integrity, authenticity, and humility bidirectionally.

Trustworthiness should be a professional competency in research. Research ethics would support this competency as qualities and attributes that sustain the values associated with ethical conduct. As we are aware of research, ethical violations, and human rights atrocities conducted in the name of medical advancement, we would like to offer the following: Participants believed most medical research would not lie to people to try to convince them to participate in research, but there was statistical significance between historically marginalized groups and white participants. The results also revealed the differences between groups regarding the following statements: “Some medical research projects are secretly designed to expose minority groups to diseases” and “Medical researchers act different toward minorities.” There are practical implications to this study and the first one is to deconstruct these statements through what we do to communicate, educate, and recruit. This requires acknowledging that some participants indeed believe the secret exposure to disease, and we must elevate the truth. Moreover, a very public case in the state of Indiana demonstrated for many that HCOs treat minoritized groups differently, even their own providers. The 2020 case of Dr Susan Moore outraged and renewed calls to address biased medical treatment of Black patients. Backed by extensive research suggesting that Black patients often receive treatment inferior to their white counterparts [25]. This prompted minoritized patients to talk about their own experiences, their grandmothers, aunt, and friends navigating the health system. They offered examples of mistreatment and questioned provider bias. As part of an exercise in critical reflexivity, providers must consider how that treatment impacts patients and research participants, and a research infrastructure aiming to improve health outcomes and the eradication of health disparities. It is what happened in history, but also what happened yesterday at their provider appointment.

In addition, the power of reflexivity cannot be underestimated. Reflexivity, the examination of one’s own beliefs and how these influence researchers, is a crucial part of practicing cultural safety and humility in research. Cultural safety and cultural humility, both significant frameworks in research require a lifelong commitment to self-evaluation and critique, redressing power imbalances, and the development of mutually beneficial partnerships with communities, while cultural safety also requires an understanding of the sociopolitical realities of those same communities included in research [26–28].

At a systems level, research institutions and research-centered organizations can also regularly ask why they are deserving of the public’s trust. In reflecting on this they will identify what researchers are asking people to trust them with and why; what accountability practices are in place and how well they work. Yarborough suggested the “deserved trust” concept to support the public’s trust, which considers what it is that the biomedical research community should be trusted to do and equip researchers and research institutions to assure that the public’s trust in their research is deserved rather than misplaced [20].

The conceptual framework contributes toward the critical examination of this study and demands that we look beyond building trust to effectively question systems we have created ourselves, and are operating in and against. A critical health lens would recognize the importance of biomedical research and how at times, it might be one of the only venues to healthcare access for sectors of our US population. In addition, the information provided throughout this article shall not just expand the valuable work in community engagement, but how essential research registries are to addressing health disparities and health outcomes.

Moreover, future work should not stop in the measurement of trust. Future research shall look at the trustworthiness of researchers and providers from their perspectives. In addition, this study provided crucial information on the perceptions of trust by historically marginalized groups, as such we will consider using scales that get to the heart of trustworthiness in diverse racial and ethnic groups. Lastly, research should look at the general population measurements, as well as more specific to participants within other research-centered aspects, such as biobanks, tissue banks, etc.

### Limitations

To the best of our knowledge, this is the first study to examine trust in healthcare organizations and (medical) researchers by multiple validated scales. However, the study specifically analyzes responses by participants familiar with biomedical research. Performing this study with a sample that had already agreed to be part of a research mission highlights self-selection bias and may not be representative of the general population. Currently, there is no comparison with the state’s general population. Also, this is centered in the state of Indiana and may not be generalizable across the USA. In addition, as it is the first study of its kind, there is no longitudinal analysis of trust within the volunteer registry. Lastly, we would like to acknowledge that participants representing historically marginalized groups were combined for statistical purposes given small sample sizes across those groups and that trends between those groups were similar. For similar reasons, Likert scale values were combined into dichotomous variables. Future research shall address these limitations.

## Conclusion

Trust can be reinforced through the design and continuous improvement of equitable partnerships that involve colearning, sharing of resources, seeking community input on the best use of resources to serve their needs, community involvement in all aspects of research or health care services design and implementation, and sharing research and program results with the community [29,30].

Several factors influence an individual’s level of trust in research including culture and beliefs, educational attainment, personal and family diagnoses, experience navigating healthcare systems, and what they know about their cultural identity and communities’ experiences with research.

We conclude that physicians, health professionals, and researchers need to better understand the nature of this trust and, quite frankly, credibility loss so they can craft effective countermeasures, but also practices that are very intentional about bringing facts and truth back to science and medicine while collaborating with community leaders to educate the public. We reaffirm the importance of understanding our communities’ trust in medical research, how we must critically assess our own trustworthiness, and critically reflect on the authenticity of our efforts.

## Study Highlights

### What is the current knowledge on the topic?

Several factors influence an individual’s level of trust in healthcare organizations and biomedical research including culture and beliefs, educational attainment, personal and family diagnoses, lived experiences, among others. Scholars and practitioners in community engagement have provided frameworks and resources emphasizing the importance of building trust.

### What question did this study address?

This study measures trust in biomedical research and healthcare organizations among individuals in a volunteer research registry and evaluates how its findings may improve recruitment as an organization.

### What does this study add to our knowledge?

This is the first study combining 3 trust scales in one survey, the Relationship of Trust in Research and Engagement survey. The survey offered an effective way to measure trust in biomedical research and healthcare organizations and provided a unique perspective of individuals whose trust in research was enough to willingly join a research registry.

### How might this change clinical pharmacology or translational science?

Researchers need to better understand the nature of this trust and implement practices that are very intentional about bringing facts and truth back to science and medicine while collaborating with community leaders to educate the public.
